# Sensitive and Rapid UHPLC-MS/MS for the Analysis of Tomato Phenolics in Human Biological Samples

**DOI:** 10.3390/molecules201119702

**Published:** 2015-11-16

**Authors:** Miriam Martínez-Huélamo, Sara Tulipani, Olga Jáuregui, Palmira Valderas-Martinez, Anna Vallverdú-Queralt, Ramón Estruch, Xavier Torrado, Rosa M. Lamuela-Raventós

**Affiliations:** 1 Department of Nutrition and Food Science-XARTA-INSA, School of Pharmacy, University of Barcelona, Barcelona 08028, Spain; mmartinezh@ub.edu (M.M.-H.); xaviertorrado@ub.edu (X.T.); 2 Centre for Biomedical Network Research on the Pathophysiology of Obesity and Nutrition (CIBEROBN), Carlos III Health Institute, Madrid 28029, Spain; palmi.vm@gmail.com (P.V.-M.); avallverdu@ub.edu (A.V.-Q.); restruch@clinic.ub.es (R.E.); 3 Biomedical Research Institute (IBIMA), Service of Endocrinology and Nutrition, Hospital Virgen de la Victoria, Teatinos Campus, University of Malaga, Malaga 29010, Spain; sara.tulipani@gmail.com; 4 Scientific and Technological Centers of the University of Barcelona (CCiTUB), Barcelona 08028, Spain; ojauregui@ccit.ub.edu; 5 Department of Internal Medicine, Hospital Clinic, Institute of Biomedical Investigation August Pi i Sunyer (IDIBAPS), University of Barcelona, Barcelona 08036, Spain; 6 INRA, UMR1083 Sciences for Oenology, 2 place Pierre Viala, Montpellier Cedex 34060, France

**Keywords:** polyphenol metabolites, validation, tomato, sauce, microbiota, metabolites, biosamples, blood, urine

## Abstract

An UHPLC-MS/MS method for the quantification of tomato phenolic metabolites in human fluids was optimized and validated, and then applied in a pilot dietary intervention study with healthy volunteers. A 5-fold gain in speed (3.5 min of total run); 7-fold increase in MS sensitivity and 2-fold greater efficiency (50% peak width reduction) were observed when comparing the proposed method with the reference-quality HPLC-MS/MS system, whose assay performance has been previously documented. The UHPLC-MS/MS method led to an overall improvement in the limits of detection (LOD) and quantification (LOQ) for all the phenolic compounds studied. The recoveries ranged between 68% and 100% in urine and 61% and 100% in plasma. The accuracy; intra- and interday precision; and stability met with the acceptance criteria of the AOAC International norms. Due to the improvements in the analytical method; the total phenolic metabolites detected in plasma and urine in the pilot intervention study were 3 times higher than those detected by HPLC-MS/MS. Comparing with traditional methods; which require longer time of analysis; the methodology described is suitable for the analysis of phenolic compounds in a large number of plasma and urine samples in a reduced time frame.

## 1. Introduction

Tomato (*Solanum lycopersicum*) is a food very rich in bioactive compounds such as vitamins or carotenoids and it contains a variety of phenolic compounds [1]. Phenolics play an important protective role in human health, decreasing mortality [2], cardiovascular disease [3], and DNA oxidation [4], and counteracting age-related cognitive decline [5]. The most important phenolics described in tomato and tomato by-products belong to hydroxycinnamic acids, flavanones and flavonols, specifically, naringenin, rutin or 5-caffeoylquinic acid [6,7]. After ingestion, polyphenols are metabolized in the small intestine and liver producing a series of metabolites (methyl, glucuronide, and sulfate), which may pass into the blood stream, accumulate in tissues, and then excreted in urine [8].

An exhaustive identification of polyphenols in food and biological samples is of great interest due to their health-promoting effects. Although a wide range of methods have been reported for the detection of phenolic compounds in food, beverages, or biological samples (*i.e*., spectrophotometry, capillary electrophoresis, near-infrared spectroscopy, HPLC-UV-DAD) [9,10,11,12], liquid chromatography coupled to mass spectrometry (LC-MS) [13,14,15,16,17,18] is the most commonly used technique due to its high sensitivity and selectivity.

In particular, ultra-high performance liquid chromatography (UHPLC) enhances chromatographic separation by using columns packed with smaller particles (1.7 µm), which provides higher resolution and better efficiency than conventional chromatography [19]. It also offers compatibility with high back-pressure operating conditions (up to 15,000 psi), which in turn increases mobile phase viscosity and the capacity to dissolve analytes [12]. The resulting higher peak resolution and shorter run times translates into lower analytical costs, and is more “environmentally friendly” in terms of reduced generation of hazardous chemical waste [20].

The aim of this work was to improve the efficiency and resolution of the HPLC-ESI-QqQ MS/MS method validated previously by our research group [21] for the detection and quantification of 5 hydroxycinnamic acids, 4 hydroxyphenylacetic acids, 2 hydroxybenzoic acids, 1 hydroxyphenylpropionic acid, 1 flavanone and 2 flavonols. Thus a new UHPLC-MS/MS-driven method was developed which involved the optimization of the main LC (column, elution solvents and gradients) and MS operating conditions (declustering potential (DP), focusing potential (FP) and collision energy (CE)), followed by its application in a pilot tomato sauce dietary intervention study.

## 2. Results

### 2.1. UHPLC-MS/MS Method Development

Table 1 shows the LOD and LOQ obtained for each compound when the study of the optimum mobile phase was achieved. Homovanillic acid, phenylacetic acid, quercetin and quercetin-3-*O*-glucuronide up to 100 ng/mL were not detected using 0.1% and 0.05% formic acid, whereas 0.025% formic acid allowed the detection and quantification of all the target compounds. H_2_O (0.025% formic acid)/MeCN (0.025% formic acid) improved the LOD of 3-hydroxyphenylacetic acid, dihydrocaffeic acid, ferulic acid, and above all isoferulic acid.

Declustering potential (DP), focusing potential (FP), entrance potential (EP), quantification and confirmation transitions with their corresponding collision energy (CE) were shown in Table 2 obtaining the optimum value for the mass conditions.

The flow rate, which allowed a correct resolution of the compounds, was achieved with 600 µL/min of H_2_O (0.025% formic acid)/MeCN (0.025% formic acid) and the best volume injection was obtained with 10 µL.

**Figure 1:**
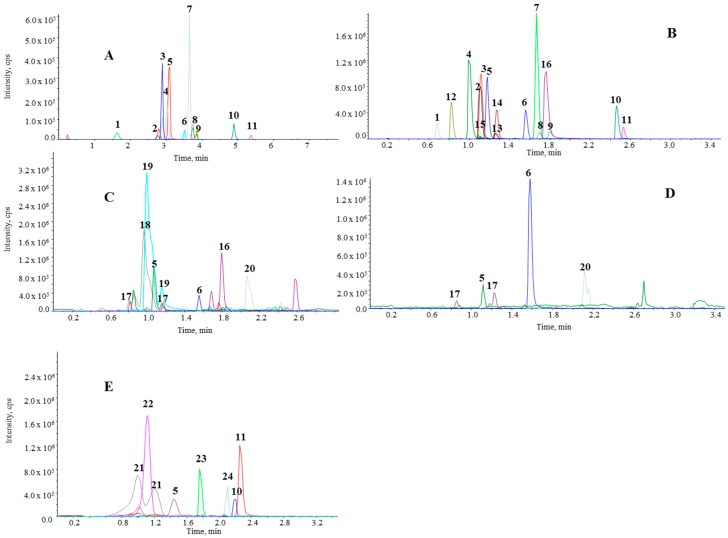
Chromatograms of standards solution obtained by HPLC-MS/MS (**A**) and UHPLC-MS/MS (**B**) analysis. Chromatograms of urine at 6 h (**C**), plasma at 1 h (**D**) and tomato sauce (**E**). Peaks: (1) 4-hydroxyhippuric acid; (2) hippuric acid; (3) dihydrocaffeic acid; (4) 5-caffeoylquinic acid; (5) caffeic acid; (6) ethylgallate (IS); (7) 3-(3-hydroxyphenyl)propionic acid; (8) ferulic acid; (9) isoferulic acid; (10) quercetin; (11) naringenin; (12) 3,4-dihydroxyphenylacetic acid; (13) homovanillic acid; (14) 3-hydroxyphenylacetic acid; (15) phenylacetic acid; (16) quercetin-3-*O*-glucuronide; (17) ferulic acid glucuronide; (18) caffeic acid sulfate; (19) ferulic acid sulfate; (20) naringenin glucuronide; (21) caffeic acid hexoside; (22) homovanillic acid hexoside; (23) rutin; (24) hydroxybenzoic acid.

Comparing a standard mix in the HPLC-MS/MS (Figure 1A) and UHPLC-MS/MS (Figure 1B) systems, the latter provided adequate resolution in less time. The UHPLC equipment allowed the quantification of 16 phenolic compounds in 3.5 min in comparison with 12 min needed for the HPLC analysis.

**Table 1:** LOD and LOQ of an aqueous mix of 15 phenolic compounds (100 ng/mL) in the study of different mobile phases by UHPLC-MS/MS.

Compounds	LOD ^a^ (ng/mL) (*n* = 3) ^c^			LOQ ^b^ (ng/mL) (*n* = 3) ^c^		
	Formic Acid (0.1%)	Formic Acid (0.05%)	Formic Acid (0.025%)	Formic Acid (0.1%)	Formic Acid (0.05%)	Formic Acid (0.025%)
Caffeic acid	1.7 ± 0.1	2.2 ± 0.1	2.1 ± 0.4	5.6 ± 0.5	7.4 ± 0.1	6.8 ± 1.3
5-Caffeoylquinic acid	0.8 ± 0.3	1.8 ± 0.3	5.3 ± 0.4	2.7 ± 0.9	6.1 ± 0.8	17.6 ± 1.3
Dihydrocaffeic acid	10.7 ± 0.6	6.9 ± 0.4	6.0 ± 0.2	35.7 ± 1.6	23.1 ± 0.9	19.8 ± 0.7
3,4-Dihydroxyphenylacetic acid	2.8 ± 0.3	8.2 ± 0.3	2.3 ± 0.4	9.5 ± 0.8	27.3 ± 1.1	7.5 ± 1.5
Ferulic acid	5.5 ± 0.6	1.8 ± 0.2	1.9 ± 0.4	18.3 ± 1.8	5.9 ± 0.7	6.2 ± 1.1
Hippuric acid	12.5 ± 1.7	9.6 ± 2.5	9.4 ± 0.9	41.5 ± 5.8	32.0 ± 2.1	31.5 ± 2.5
Homovanillic acid	n.d. ^d^	n.d. ^d^	49.2 ± 2.4	n.d. ^d^	n.d. ^d^	163.9 ± 6.8
4-Hydroxyhippuric acid	0.6 ± 0.1	1.3 ± 0.2	2.5 ± 0.4	2.1 ± 0.6	4.2 ± 0.8	8.3 ± 1.4
3-Hydroxyphenylacetic acid	12.0 ± 1.3	7.1 ± 0.3	6.6 ± 0.6	39.8 ± 3.6	23.7 ± 0.7	21.8 ± 2.1
3-(3-Hydroxyphenyl)propionic acid	5.7 ± 0.9	6.6 ± 0.2	3.4 ± 0.1	18.9 ± 2.7	22.0 ± 0.5	11.2 ± 0.5
Isoferulic acid	54.6 ± 2.2	5.9 ± 0.1	8.9 ± 0.9	181.8 ± 8.4	19.6 ± 0.4	29.7 ± 3.3
Naringenin	0.5 ± 0.1	0.4 ± 0.2	0.7 ± 0.4	1.6 ± 0.4	1.2 ± 0.8	2.4 ± 1.1
Phenylacetic acid	n.d. ^d^	n.d. ^d^	35.3 ± 3.0	n.d. ^d^	n.d. ^d^	117.7 ± 8.9
Quercetin	n.d. ^d^	n.d. ^d^	21.9 ± 0.9	n.d. ^d^	n.d. ^d^	73.0 ± 2.3
Quercetin-3-*O*-glucuronide	n.d. ^d^	n.d. ^d^	22.4 ± 0.2	n.d. ^d^	n.d. ^d^	74.6 ± 0.2

Values are means ± standard deviation; LOD ^a^: limit of detection; LOQ ^b^: limit of quantification; (*n* = 3) ^c^: replicates; n.d. ^d^: no detected.

**Table 2:** Rt (Retention time), Declustering potential (DP), focusing potential (FP) and entrance potential (EP) optimized. Quantification and confirmation transitions of the phenolic compounds with the optimum collision energy (V).

Compounds	Rt (Min)	DP (V)	FP (V)	EP (V)	Quantification Transition	Collision Energy (V)	Confirmation Transition	Collision Energy (V)
Caffeic acid	1.18	−40	−170	−10	179 → 135	−20	179 → 107	−30
5-Caffeoylquinic acid	1.01	−50	−180	−10	353 → 191	−20	353 → 179	−30
Dihydrocaffeic acid	1.12	−50	−170	−10	181 → 137	−20	181 → 121	−30
3.4-Dihydroxyphenylacetic acid	0.83	−40	−170	−10	167 → 123	−10		
Ethylgallate (IS)	1.56	−60	−200	−10	197 → 169	−25	197 → 124	−40
Ferulic acid	1.70	−40	−170	−10	193 → 134	−20	193 → 178	−30
Hippuric acid	1.10	−40	−170	−10	178 → 134	−20		
Homovanillic acid	1.27	−40	−170	−10	181→ 137	−20		
4-Hydroxyhippuric acid	0.68	−40	−170	−10	194 → 100	−20	194 → 150	−30
3-Hydroxyphenylacetic acid	1.28	−40	−170	−10	151 → 107	−10		
3-(3-Hydroxyphenyl)propionic acid	1.67	−40	−170	−10	165 → 121	−20	165 → 119	−35
Isoferulic acid	1.80	−50	−220	−10	193 → 178	−20	193 → 134	−35
Naringenin	2.51	−50	−190	−10	271 → 151	−30	271 → 119	−40
Phenylacetic acid	1.10	−50	−170	−10	135 → 91	−30		
Quercetin	2.46	−60	−210	−10	301 → 151	−30	301 → 179	−40
Quercetin-3-*O*-glucuronide	1.76	−60	−210	−10	477 → 301	−30	477 → 151	−40

### 2.2. Validation Parameters

#### 2.2.1. Limits of Detection (LOD) and Quantification (LOQ)

The sensitivity of the method was evaluated by determining the LOD and LOQ. There were no differences in LOD and LOQ values between the two biosamples (urine and plasma) (Table 3). Nevertheless, the results showed a wide range of sensitivity according to the analyte. LOD were established between 0.5 and 62.5 ng/mL in urine and 0.3 and 44.1 ng/mL in plasma. In the case of LOQ, values ranged from 1.8 to 203.4 ng/mL in urine and 1.6 to 145.8 ng/mL in plasma. The most notable improvement was for 4-hydroxyhippuric acid and isoferulic acid, whose LOD decreased 25-fold and 15-fold in urine and 11-fold and 9-fold in plasma, respectively if HPLC and UHPLC were compared. The LOD for ferulic and hippuric acids also decreased, particularly in plasma, but to a lesser extent.

**Table 3:** LOD, LOQ, recovery, concentration range and correlation coefficient in urine and plasma samples by HPLC-MS/MS and UHPLC-MS/MS.

Compounds	HPLC (*n* = 3) ^a^				UHPLC (*n* = 3) ^a^				HPLC (*n* = 3) ^a^		UHPLC (*n* = 3) ^a^		HPLC		UHPLC	
	URINE		PLASMA		URINE		PLASMA		URINE	PLASMA	URINE	PLASMA	URINE	PLASMA	URINE	PLASMA
	LOD ^b^ (ng/mL)	LOQ ^c^ (ng/mL)	LOD ^b^ (ng/mL)	LOQ ^c^ (ng/mL)	LOD ^b^ (ng/mL)	LOQ ^c^ (ng/mL)	LOD ^b^ (ng/mL)	LOQ ^c^ (ng/mL)	Rec. ^e^ (%)	Rec. ^e^ (%)	Rec. ^e^ (%)	Rec. ^e^ (%)	Conc. Range ^f^ (ng/mL) (r^2^) ^g^	Conc. Range ^f^ (ng/mL) (r^2^) ^g^	Conc. Range ^f^ (ng/mL) (r^2^) ^g^	Conc. Range ^f^ (ng/mL) (r^2^) ^g^
Caffeic acid	1.5 ± 0.1	6.0 ± 0.1	1.8 ± 0.1	6.0 ± 0.4	0.5 ± 0.2	2.0 ± 0.4	0.7 ± 0.1	2.8 ± 0.1	97 ± 4	98 ± 3	100 ± 2	91 ± 2	6–3450 (0.990)	6–3450 (0.993)	2–1152 (0.994)	3–1728 (0.995)
5-Caffeoylquinic acid	0.5 ± 0.1	2.0 ± 0.2	0.6 ± 0.1	2.0 ± 0.1	1.1 ± 0.1	4.3 ± 0.1	0.5 ± 0.1	1.7 ± 0.1	103 ± 2	99 ± 4	99 ± 4	92 ± 2	2–1150 (0.999)	2–1150 (0.999)	4–2304 (0.992)	2–1152 (0.998)
Dihydrocaffeic acid	6.3 ± 0.2	25 ± 0.9	4.4 ± 0.1	15 ± 0.3	5.7 ± 0.6	20.2 ± 2.1	5.3 ± 0.3	18.0 ± 1.0	101 ± 2	104 ± 5	97 ± 5	90 ± 4	25–14,400 (0.993)	15–14,400 (0.991)	20–11,520 (0.996)	18–10,368 (0.992)
3,4-Dihydroxyphenylacetic acid	n.s. ^d^	n.s. ^d^	n.s. ^d^	n.s. ^d^	1.1 ± 0.1	3.5 ± 0.2	1.6 ± 0.1	5.4±0.2	n.s. ^d^	n.s. ^d^	78 ± 3	69 ± 2	n.s. ^d^	n.s. ^d^	4–2304 (0.994)	5–2880 (0.998)
Ferulic acid	15 ± 0.7	50 ± 2.7	18 ± 2.1	60 ± 7.0	1.9 ± 0.1	6.6 ± 0.2	1.6 ± 0.1	5.6 ± 0.2	95 ± 2	98 ± 4	100 ± 4	100 ± 3	50–28,800 (0.990)	60–28,800 (0.996)	7–4032 (0.998)	6–3456 (0.990)
Hippuric acid	25 ± 1.6	80 ± 3.2	25 ± 0.8	90 ± 2.1	4.4 ± 0.2	15.0 ± 0.6	2.1 ± 0.3	6.5 ± 0.9	106 ± 2	99 ± 4	97 ± 2	99 ± 4	80–51,840 (0.990)	90–51,840 (0.992)	15–8640 (0.999)	7–4032 (0.991)
Homovanillic acid	n.s. ^d^	n.s. ^d^	n.s. ^d^	n.s. ^d^	4.5 ± 0.1	17.3 ± 0.4	8.5 ± 0.3	29.2 ± 1.0	n.s. ^d^	n.s. ^d^	97 ± 4	92 ± 4	n.s. ^d^	n.s. ^d^	17–9792 (0.996)	29–16,704 (0.991)
4-Hydroxyhippuric acid	15 ± 1.2	50 ± 2.6	15 ± 0.7	50 ± 3.2	0.6 ± 0.1	1.8 ± 0.2	1.4 ± 0.1	4.6 ± 0.3	75 ± 3	73 ± 3	68 ± 3	61 ± 4	50–28,800 (0.990)	50–28,800 (0.992)	2–1152 (0.999)	5–2880 (0.999)
3-Hydroxyphenylacetic acid	n.s. ^d^	n.s. ^d^	n.s. ^d^	n.s. ^d^	0.9 ± 0.1	2.8 ± 0.2	1.6 ± 0.3	5.3 ± 0.9	n.s. ^d^	n.s. ^d^	99 ± 2	98 ± 2	n.s. ^d^	n.s. ^d^	3–1728 (0.999)	5–2880 (0.998)
3-(3-Hydroxyphenyl)propionic acid	6.0 ± 0.8	20 ± 1.4	6.0 ± 1.2	20 ± 2.9	2.5 ± 0.1	9.0 ± 0.2	2.4 ± 0.2	8.6 ± 0.5	97 ± 2	99 ± 5	98 ± 4	94 ± 3	20–11,520 (0.995)	20–11,520 (0.994)	9–5184 (0.991)	9–5184 (0.994)
Isoferulic acid	29 ± 4.3	90 ± 13.6	30 ± 2.4	105 ± 8.0	2.0 ± 0.3	6.2 ± 1.0	3.4 ± 0.2	12.0 ± 0.5	99 ± 2	99 ± 4	100 ± 3	97 ± 4	90–28,800 (0.997)	105–28,800 (0.994)	6–3456 (0.993)	12–6912 (0.993)
Naringenin	0.5 ± 0.1	2.0 ± 0.1	0.5 ± 0.1	2.0 ± 0.1	0.6 ± 0.1	2.8 ± 0.3	0.3 ± 0.1	1.6 ± 0.1	104 ± 4	96 ± 3	99 ± 2	96 ± 2	2–1150 (0.999)	2–1150 (0.995)	3–1728 (0.999)	2–1152 (0.999)
Phenylacetic acid	n.s. ^d^	n.s. ^d^	n.s. ^d^	n.s. ^d^	62.5 ± 3.0	203.4 ± 9.9	44.1 ± 5.4	145.8 ± 17.8	n.s. ^d^	n.s. ^d^	95 ± 3	99 ± 4	n.s. ^d^	n.s. ^d^	203–116,929 (0.992)	146–84,096 (0.994)
Quercetin	1.7 ± 0.1	6.0 ± 0.3	1.4 ± 0.1	5.0 ± 0.4	2.3 ± 1.4	8.5 ± 4.8	4.5 ± 0.1	15.8 ± 0.3	65 ± 3	100 ± 3	85 ± 4	100 ± 2	6–3450 (0.991)	5–3450 (0.990)	9–5184 (0.998)	16–9216 (0.998)
Quercetin-3-*O*-glucuronide	n.s. ^d^	n.s. ^d^	n.s. ^d^	n.s. ^d^	1.4 ± 0.1	4.4 ± 0.1	1.9 ± 0.1	6.1 ± 0.2	n.s. ^d^	n.s. ^d^	99 ± 2	95 ± 2	n.s. ^d^	n.s. ^d^	5–2880 (0.997)	6–3456 (0.993)

Values are means ± standard deviation; (*n* = 3) ^a^: replicates; LOD ^b^: limit of detection; LOQ ^c^: limit of quantification; n.s. ^d^: not studied; Rec. ^e^: recovery; Conc. Range ^f^: concentration range; (r^2^) ^g^: correlation coefficient.

#### 2.2.2. Linearity

Calibration curves showed linear responses between LOQ and 576 times LOQ for each analyte, establishing ranges from 2 ng/mL to 116,929 ng/mL in urine, and 2 ng/mL to 84,096 ng/mL in plasma. A weighting factor was necessary for almost all the phenolic compounds studied to achieve accuracy between 85%–115%. The correlation coefficient (r^2^) between 0.990 and 0.999 for plasma and 0.991 and 0.999 for urine demonstrate an adequate linearity, similarly of those obtained in the HPLC-MS/MS validation [21].

#### 2.2.3. Recovery

Table 3 shows the recoveries obtained with UHPLC system and the previous HPLC method validation. In the HPLC system, the recovery of the compounds studied ranged from 65% to 106% in urine and 73% to 104% in plasma. The lowest recovery in urine was for quercetin, and 4-hydroxyhippuric acid in plasma. In the case of UHPLC, there was little variation between the two biological matrices, with recoveries greater than 85% except for 3,4-dihydroxyphenylacetic acid and 4-hydroxyhippuric acid (78% in urine and 69% in plasma, and 68% in urine and 61% in plasma, respectively).

#### 2.2.4. Accuracy and Precision

Intra- and interday accuracy and precision were studied by injection of plasma and urine extracts spiked at three different concentrations: low (3-fold the LOQ), medium (48-fold the LOQ), and high (288-fold the LOQ). The accuracy obtained was between 91.3% and 113.9% in urine, and 99.0% and 114.8% in plasma, thereby meeting with the AOAC acceptance criteria. Phenylacetic acid was the phenolic acid in urine with both the highest intra- and interday accuracy. In plasma, the top percentage was given by homovanillic acid. Intra- and interday precision studies gave the same results as for accuracy. No polyphenol in urine or plasma samples exceeded the 15% RSD set by the AOAC. The highest values were 3.6% RSD in urine, corresponding to 4-hydroxyhippuric acid, and 13.5% RSD in plasma, to quercetin.

#### 2.2.5. Stability

The processed sample stability refers to the variation of concentration, since the SPE is performed until the samples are in the autosampler, in this case about 24 h. The results show a reduction in concentration ranging between 3.0% for caffeic acid and 12.9% for phenylacetic acid in urine, and 2.8% for homovanillic acid and 13.3% for quercetin in plasma, but without significant differences between the biological samples and standards. For the freeze/thaw stability, three freeze-thaw cycles were assessed for a mix of the standards used for the validation in plasma and urine samples. Similar results were obtained, with a reduction ranging from 3.1% for dihydrocaffeic acid to 12.6% for quercetin in urine, and 3.8% for 3-hydroxyphenylacetic acid to 9.1% for quercetin in plasma.

### 2.3. Phenolic Quantification in Tomato Sauce and Biological Samples

**Figure 2:**
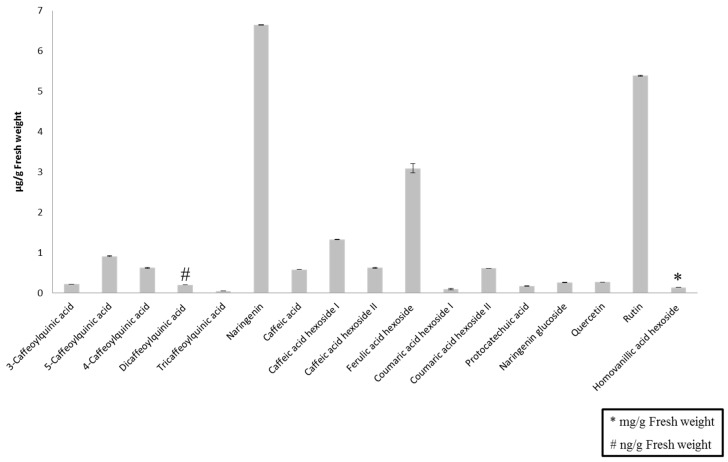
Quantification of phenolic compounds from tomato sauce administered to the volunteers by UHPLC-MS/MS analysis. Values are mean ± standard deviation (µg/g FW). Diccaffeoylquinic acid was expressed as ng/g FW and homovanillic acid hexoside as mg/g FW.

Figure 2 shows the concentration of the phenolic compounds in the tomato sauce administered to the eight volunteers. The main polyphenols present in the tomato sauce were from two classes: flavonoid and phenolic acids, the latter represented by a wider range of compounds (mono-, di- and tricaffeoylquinic acids, caffeic acid and two hexosides, ferulic acid hexoside, two coumaric acid hexosides, protocatechuic acid, and homovanillic acid hexoside). Homovanillic acid hexoside was the predominant polyphenol in the sauce (0.140 ± 0.005 mg/g FW) highlighting above the rest of compounds. Flavanones and flavonols were also found in the tomato sauce being naringenin and rutin the compounds with higher concentration, 6.650 ± 0.003 and 5.390 ± 0.015 µg/g FW, respectively.

Table 4 shows the concentration of phenolic compounds found in plasma and urine after the tomato sauce intervention. Twelve phenolic compounds were quantified in plasma and twenty-eight in urine by UHPLC-MS/MS. The concentration ranged between 2.7 to 183 ng/mL in plasma for 5-caffeoylquinic acid and caffeic acid sulfate, respectively, and between 17.0 to 33,188 ng/mL for 5-caffeoylquinic acid and 4-hydroxyhippuric acid, respectively. A great variety of metabolites belonging to microbiota were quantified in urine at high concentrations as 16,425 ng/mL for 3-hydroxyphenylacetic acid sulfate or 225,696 ng/mL for ferulic acid sulfate.

**Table 4:** Phenolic compounds and metabolites quantified in urine and plasma by UHPLC-MS/MS.

Compounds	Product Ions in MS^2^ Experiments	Urine (ng/mL)	Plasma (ng/mL)
Caffeic acid *	179,135,107	206 (4.0–822)	n.d. ^a^
Caffeic acid glucuronide (CA)	355,179,135,175,113	54.9 (2.0–975)	6.4 (3.0–12.1)
Caffeic acid sulfate (CA)	259,179,135	1396 (10.2–42,286)	183 (15.3–862)
5-Caffeoylquinic acid *	353,191,179	17.0 (7.2–53.6)	2.7 (2.0–3.5)
Dihydrocaffeic acid *	181,137,59	194.7 (30.1–1031.9)	n.d. ^a^
Dihydrocaffeic acid glucuronide (DHCA)	357,181,137,175,113	2127 (23–10,071)	n.d. ^a^
Dihydrocaffeic acid sulfate (DHCA)	261,181,137	2775 (52–9424)	n.d. ^a^
3,4-Dihydroxyphenylacetic acid *	167,123	1095 (33.9–2249)	n.d. ^a^
Ferulic acid *	193,134,175	453 (31–2139)	8.1 (6.5–40.3)
Ferulic acid glucuronide (FA)	369,193,134,175,113	2852 (110–69,495)	53.5 (6.7–1881)
Ferulic acid sulfate (FA)	273,193,134	22,569 (713–536,479)	95.5 (24.5–316)
Homovanillic acid *	181,137	4050 (1073–9387)	n.d. ^a^
4-Hydroxyhippuric acid *	194,100	33,188 (691–214,695)	n.d. ^a^
3-Hydroxyphenylacetic acid *	151,107	739 (244–1291)	n.d. ^a^
3-Hydroxyphenylacetic acid glucuronide (3-HPAA)	327,151,107,175,113	295 (11.0–1608)	n.d. ^a^
3-Hydroxyphenylacetic acid sulfate (3-HPAA)	231,151,107	16,425 (175–352,967)	n.d. ^a^
3-(3-Hydroxyphenyl)propionic acid *	165,121	1402 (188–4007)	n.d. ^a^
3-(3-Hydroxyphenyl)propionic acid glucuronide (3-(3-HPPA))	341,165,121,175,113	258 (15.3–4914)	n.d. ^a^
3-(3-Hydroxyphenyl)propionic acid sulfate (3-(3-HPPA))	245,165,121	3156 (27.6–145,199)	n.d. ^a^
Isoferulic acid *	193,134,175	1156 (247–3427)	108 (10.6–494)
Naringenin *	271,151,119	39.1 (6.9–400)	11.7 (2.0–52.6)
Naringenin glucuronide (N)		854 (214–1558.5)	73.4 (2.0–830)
Phenylacetic acid *	135,91	1129 (297–6667)	n.d. ^a^
Phenylacetic acid glucuronide (PAA)	311,135,91,175,113	318 (206–431)	n.d. ^a^
Phenylacetic acid sulfate (PAA)	215,135,91	1378 (110–26,708)	n.d. ^a^
Quercetin *	301,151,179	70.8 (9.1–417)	99.0 (23.7–331)
Quercetin glucuronide *	477,301,151,175,113	14.4 (11.9–101)	20.9 (14.3–63.4)
Quercetin sulfate (Q)	381,301,151	32.0 (11.9–489.8)	3.8 (3.5–4.2)

Values are median (minimum-maximum values); *: commercial standard; n.d. ^a^: not detected; CA: Caffeic Acid; DHCA: Dihydrocaffeic Acid; FA: Ferulic Acid; 3-HPAA: 3-Hydroxyphenylacetic acid; 3-(3-HPPA): 3-(3-Hydroxyphenyl)propionic acid; N: Naringenin; PAA: Phenylacetic acid; Q: Quercetin.

## 3. Discussion

### 3.1. UHPLC-MS/MS Method Development

Before the validation of the method, different mobile phases were tested to obtain the best detection and separation of the compounds (Table 1). Between the three phases studied, 0.1%, 0.05% and 0.025% of formic acid, the mobile phase with less formic acid achieved the best results confirming that low-pH conditions created by high acid concentrations (up to 0.1%) decrease the negative-ion ESI response by damaging the formation of the deprotonated analyte [22].

After the election of the mobile phase, several experiments with 50:50 (*v*/*v*) of (0.025% formic acid)/MeCN (0.025% formic acid) were achieved to obtain the optimum DP, FP, EP, CE and the quantification and confirmation transitions which are shown in Table 2. The results were similar as those obtained in a previous validation method developed in HPLC-MS/MS [21].

Different linear gradient were proved to attain a good separation and resolution of the analytes. The best results obtained for the conditions were described in the chromatographic separation section with a total time run of 3.5 min. Diverse flow rates, 400, 600 and 800 µL/min, and injection volumes, 2, 5 and 10 µL were also studied to obtain a good separation and detection of the analytes. The optimum flow rate was achieved in 600 µL/min, as in the HPLC-MS/MS method. 10 µL was demonstrated to be the best injection volume to obtain the greatest detected peak height. The injection volume was halved regarding to the HPLC-MS/MS method allowing more volume sample if various injections were needed.

The time-saving and decrease in the total consumption of mobile phase implies a reduction in analysis cost [20,23]. Compared with HPLC-MS/MS [21], the UHPLC-MS/MS method offered a 5-fold decrease in retention time (RT), up to 7-fold increase in detected peak height, and a 2-fold decrease in peak width thereby enhancing the sensitivity of the method. 

### 3.2. Validation Parameters

The AOAC International criteria were consulted as to the validation of the method [24]. A great improvement in terms of LOD and LOQ was achieved using UHPLC compared with HPLC for almost all the studied phenolic compounds, in both plasma and urine (Table 3). In comparison with our work, Rubió *et al.* [25] obtained higher LOD (1–240 ng/mL) and LOQ (3.3–801.5 ng/mL) in the validation of an UHPLC-MS/MS system to detect plasma phenolic metabolites. In particular, our method performed better for caffeic acid, with a LOD of 0.7 ng/mL compared to 64.9 in the study of Rubió *et al.* [25]. 3,4-Dihydroxyphenylacetic acid and 3-(3-hydroxyphenyl)propionic acid followed the same trend as caffeic acid, with lower values in our study (1.6 and 2.4 ng/mL, respectively), compared to the data of Rubió *et al.* [25] (15.1 and 38.2 ng/mL, respectively). Another study, carried out by Magiera *et al.* [16] to determine polyphenols and their metabolites in human urine by UHPLC coupled to a 4000 Q TRAP triple quadrupole linear ion trap mass spectrometer, obtained a LOQ for 3,4-dihydroxyphenylacetic acid comparable to our results, while other metabolites, for example, caffeic acid, presented lower values (1 ng/mL) in comparison with our data (2.0 ng/mL). Lastly, Oliveira *et al.* [26] reported the validation of a method that detected 11 phenolic acids in plasma, urine and liver by an UHPLC system coupled to a single-quadrupole mass spectrometer. The LOQ for caffeic, ferulic and 5-caffeoylquinic acids were 38, 52 and 52 ng/mL, respectively, in plasma, and 50, 96 and 48 ng/mL, respectively, in urine. Our study achieved a greater improvement in LOD in comparison with Oliveira *et al.* [26].

Recovery results were similar to those reported by Rubió *et al.* [25], who found levels of 77% for quercetin and 3,4-dihydroxyphenylacetic acid, and 98% for naringenin. Our data are also in agreement with Magiera *et al.* [16], who reported recoveries between 91% and 100% for caffeic, ferulic, and 3-hydroxyphenylacetic acids and naringenin. In another study on phenolic microbial metabolites in humans and rats, the mean recovery of analytes ranged from 87% to 109% [27]. Our results are therefore in agreement with the reported literature data.

Respect to accuracy and precision, a possible matrix effect may explain the higher values for quercetin and 4-hydroxyhippuric acid, as peaks with good symmetry were obtained without tailing, as can be seen in Figure 1. The proposed method therefore demonstrated good accuracy and precision in both urine and plasma samples. The results were similar to those obtained by HPLC-MS/MS, confirming that the method is applicable for the determination of phenolic compounds in both types of biological samples. The high accuracy for homovanillic acid and phenylacetic acid, although still within the limits established by the AOAC, may be due to their similar elution times, since they practically co-eluted.

Finally, quercetin seems to be the phenolic compound most affected in terms of stability, either in freeze/thaw or post-preparative studies. Ramešová *et al*. [28] reported that quercetin was potentially affected by exposure to atmospheric oxygen conditions. Their study confirmed the presence of four decomposition products by HPLC-DAD and HPLC-MS: 2-(3′,4′-dihydroxybenzoyl)-2,4,6-trihydroxybenzofuran-3(2*H*)-one, 2-(3,4-dihydroxyphenyl)-2-oxoacetic acid, 2,4,6-trihydroxybenzoic acid and 3,4-dihydroxybenzoic acid [28]. Although there was a decrease in the stability of some polyphenols, there were no significant differences in either two stability studies.

### 3.3. Phenolic Quantification in Tomato Sauce and Biological Samples

Three classes of polyphenols were quantified: 13 phenolic acids (three caffeoylquinic acids and two derivatives; caffeic acid and two hexosides; one ferulic acid hexoside; two coumaric acid hexosides; protocatechuic acid; and homovanillic acid hexoside), two flavanones (naringenin and naringenin glucoside), and two flavonols (quercetin and rutin). Most of the polyphenols belong to phenolic acids, being the homovanillic acid hexoside the major one in the tomato sauce. Naringenin and rutin were the most abundant phenolic compounds preceding homovanillic acid hexoside. Other authors have also described those as the three major polyphenols in tomato and tomato by-products [6,29,30,31]. Ferulic acid hexoside, caffeic acid hexoside I and 5-caffeoylquinic acid were also present at high concentration, similarly to those obtained by Minoggio *et al.* [32] between 300 and 5800 ng/g FW. The validated method was successfully applied for the analysis of human plasma and urine samples from the intervention study. The biological samples were screened for the phenolic compounds previously analyzed in the validation study, identifying and quantifying the analytes by comparing their MRM transition, RT, and product ion scan with those of the standards. Phase II metabolites (glucuronide and sulfate conjugates) were also monitored to shed more light on the metabolism of the target compounds. In the absence of standards, the phenolic metabolites were identified by PIS, NL or PrIS (Table 4). Samples over the calibration curve were diluted and reinjected in the UHPLC-MS/MS system. Polyphenols, when reached the intestine, are transformed in a wide variety of phenolic metabolites [33,34], that are absorbed by the gut, circulated in the blood and metabolized in the liver to glucuronides or sulfates metabolites [35]. Table 4 confirmed a great metabolism of the phenolic compounds described in the tomato sauce as 12 phenolic compounds were quantified in plasma and 28 in urine. Both, plasma and urine metabolites tripled the compounds determined by HPLC-MS/MS [36]. Notably, isoferulic acid, caffeic acid sulfate, ferulic acid sulfate, dihydrocaffeic acid metabolites and quercetin and its metabolites (glucuronide and sulfate), none of which were identified by HPLC-MS/MS in either plasma or urine, were detected by UHPLC-MS/MS.

We can therefore report an efficient performance by the validated UHPLC method, as it allowed the identification of phenolic compounds or metabolites undetected by the HPLC system, due to the improvement in the limits of detection and quantification for almost all compounds.

## 4. Experimental Section

### 4.1. Chemicals

Caffeic acid, 5-caffeoylquinic acid, dihydrocaffeic acid, 3,4-dihydroxyphenylacetic acid, ethylgallate (internal standard (IS)), ferulic acid, hippuric acid, homovanillic acid, 3-hydroxyphenylacetic acid, 3-hydroxybenzoic acid, isoferulic acid, naringenin, naringenin glucoside, *p*-coumaric acid, protocatechuic acid, quercetin-3-*O*-glucuronide and rutin were purchased from Extrasynthese (Genay, France); 3-(3-hydroxyphenyl)propionic acid, human plasma, phenylacetic acid and quercetin were supplied by Sigma-Aldrich (St Louis, MO, USA); and 4-hydroxyhippuric acid was purchased from PhytoLab GmbH & Co. KG. (Vestenbergsgreuth, Germany). The purity of all standards was superior at 90%. All reagents were of HPLC grade: ethanol (EtOH), acetonitrile (MeCN), methanol (MeOH), and *o*-phosphoric acid 85% were purchased from Panreac Quimica S.A. (Barcelona, Spain); and formic acid was from Scharlau Chemie S.A. (Barcelona, Spain). Ultrapure water (Milli-Q) was obtained from a Millipore system (Millipore, Bedford, MA, USA).

### 4.2. Method Development

#### 4.2.1. UHPLC Column

For the development of a faster chromatographic method, smaller particles and inner column diameters are needed [37,38]. A Waters BEH C_18_ column (50 mm × 2.1 mm) packed with 1.7 µm particles using an Acquity UPLC BEH C_18_ VanGuard pre-column 1.7 µm (2.1 mm × 5 mm) was selected for the development of the UHPLC method instead of the Luna C_18_ (50 mm × 2.0 mm) of 5 μm used for the analysis in the HPLC method [21].

#### 4.2.2. Mobile Phase

The method validated in the HPLC-MS/MS system was adjusted to be used in the UHPLC equipment. To obtain a better separation and resolution of the analytes, an aqueous mix of 15 commercial phenolic compounds (100 ng/mL final concentration) were analyzed, using the following mobile phase combinations: (1) [A] H_2_O (0.1% formic acid)/[B] MeCN (0.1% formic acid); (2) [A] H_2_O (0.05% formic acid)/[B] MeCN (0.05% formic acid); and (3) [A] H_2_O (0.025% formic acid)/[B] MeCN (0.025% formic acid).

#### 4.2.3. MS Conditions

An API 3000 triple-quadrupole mass spectrometer (Sciex, Framingham, MA, USA) with a turbo ion spray source controlled by Analyst v.1.4.2 software supplied by Sciex (Framingham, MA, USA, version 1.4.2) was used for infusion experiments. 50:50 (*v*/*v*) of water (0.025% formic acid)/MeCN (0.025% formic acid) was employed for infusion experiments, injecting each phenolic standard individually at a concentration of 1 μg/mL. The Turbo Ion spray source was used in negative mode with the following settings: capillary voltage, −3500 V; nebulizer gas (N_2_), 10 (arbitrary units); curtain gas (N_2_), 12 (arbitrary units); drying gas (N_2_) was heated to 400 °C and introduced at a flow rate of 5000 cm^3^/min. Table 2 shows the optimal DP, FP, and EP to enhance the ESI detection of the target phenolics. Multiple reaction monitoring (MRM) experiments in the negative ionization mode were performed using a dwell time of 30 ms, with 434 cycles and between 10 to 14 data points on the chromatographic peaks. The ions in MRM mode were produced by collision-activated dissociation (CAD) of selected precursor ions in the collision cell of the triple quadrupole and analyzed with the second analyzer of the instrument. The optimum collision-activated dissociation (N_2_) was 4 (arbitrary units). The transition chosen for the quantification and confirmation are shown in Table 2 with its appropriate CE.

#### 4.2.4. Flow Rate and Volume of Injection

Once obtained the best mobile phase and the optimum mass conditions for each analyte, several flow rates, 400, 600 and 800 µL/min, were studied at the same time that the volume of injection comparing 2, 5 and 10 µL to enhance the separation and detection of the analytes.

#### 4.2.5. Chromatographic Separation

The final mobile phase used was water (A) and MeCN (B) with 0.025% formic acid in both solvents. An increasing linear gradient of B was used (t (min), %B), as follows: (0.0, 5); (2.0, 25); (2.5, 90); (2.65, 100); (2.8, 100); (2.9, 5), and (3.5, 5). The mobile-phase flow rate for the biological samples was 600 µL/min, and 10 µL of the sample was injected into the UHPLC system.

### 4.3. Quality Parameters

The method was validated following the criteria of AOAC International [24]. The quality parameters established were LOD, LOQ, linearity, recovery, accuracy, precision, and stability.

LOD is the smallest quantity of analyte that can be shown to be significantly greater than the measurement error of the blank at a prescribed level of confidence. The LOD was estimated from the chromatograms of spiked blank plasma and urine samples at the lowest analyte concentration tested for a signal-to-noise ratio of 3. Similarly, LOQ, the smallest amount of analyte in a test sample that can be quantitatively determined with suitable precision and accuracy, was determined for a signal-to-noise ratio of 10. Spiked plasma and urine samples at five different concentration levels, ranged between 0.05 and 300 ng/mL, were prepared in triplicate in order to establish the LOD and LOQ in the different mass spectrometric systems.

The IS method was used for the preparation of the calibration curves using eight different concentrations within the range of the LOQ for each analyte to 576 times the LOQ. In order to obtain the most reliable calibration curve, a 1/x or 1/(x × x) weighting factor, or none, was applied, according to the analyte. The calculated standard concentration was established within 15% deviation from the nominal value except at the LOQ concentration, for which the maximum acceptable deviation was set at 20%.

Recovery was assessed by preparing eight-point calibration curves (pre-extracted spiked samples) and eight-point external curves (post-extracted spiked samples). To calculate recovery, concentration must first be computed by interpolating areas obtained from the post-extracted spiked samples into the pre-extracted spiked calibration curve. Then, the ratio analyte concentration/IS concentration was plotted against the calculated concentration explained above and a linear regression model was applied. The slope of the linear regression multiplied by 100 represents the analyte recovery.

Accuracy was determined by spiking blank urine and plasma with three known concentrations: low (3-fold the LOQ), medium (48-fold the LOQ), and high (288-fold the LOQ), with respect to the calibration curves, in five replicates. The results were expressed as the percentage of the ratio of the mean concentration observed and the known spiked concentration in the biological matrices. The mean value should be within 15% of the nominal value. Intra- and interday precision was assessed using five determinations per three concentration levels (low (3-fold the LOQ), medium (48-fold the LOQ), and high (288-fold the LOQ)) in a single analytical run or on three different days, respectively. The precision determined at each concentration level should not exceed 15% of the relative standard deviation (RSD).

The chemical stability of an analyte in a given matrix under specific conditions for given time intervals is assessed in several ways. Stability evaluations should cover the expected sample handling and storage conditions during the length of the study. The factors studied in this method were freeze-thaw cycle stability and processed sample stability.

### 4.4. Method Application: Pilot Dietary Intervention Study

#### 4.4.1. Biological Material

The optimized method was applied to a small-scale prospective single-arm intervention study conducted in eight volunteers aged between 19 and 38 years (28 ± 6.9 years) with a mean body mass index of 23 ± 3.73 kg/m^2^. On the day of intervention, the volunteers consumed 250 mL of tomato sauce per 70 kg of body weight. Blood was collected 1 h and urine 3–6 h after the consumption of the intervention and stored at −80 °C until analysis.

Commercial tomatoes (*Lycopersicum esculentum* L.) were used for the elaboration of the tomato sauce at the Torribera Campus, University of Barcelona (Santa Coloma de Gramanet, Barcelona) following a standardized making process [36].

The study protocol was approved by the Ethics Committee of Clinical Investigation of the University of Barcelona (Spain), and the clinical trial was registered at the International Standard Randomized Controlled Trial Number (ISRCTN20409295). Informed consent was obtained from all participants.

#### 4.4.2. Phenolics of Tomato Sauce and Biological Samples Extraction

A liquid-liquid extraction with ethanol/H_2_O (0.1% formic acid) (80/20, *v*/*v*) was used to extract the phenolic compounds from the tomato sauce, as previously described by Di Lecce *et al.* [7]. Briefly, tomato sauce (0.3 g) was weighed and ethanol/H_2_O (0.1% formic acid) (80/20, *v*/*v*, 3 mL) added. The homogenate was sonicated for 5 min and centrifuged at 4000 rpm for 20 min at 4 °C. The supernatant was collected, and the extraction procedure was repeated. Both supernatants were combined and the ethanol phase evaporated under a stream of nitrogen gas. The residues were reconstituted up to 1.2 mL with water containing 0.1% formic acid, filtered thought a 0.22 μm polytetrafluoroethylene (PTFE) syringe filters (Waters Corporation, Mildfore, MA, USA), and injected into the UHPLC-MS/MS system. Extractions were performed in triplicate and quantified with the corresponding commercial standards. When standards were not available, as in the case of di-, tricaffeoylquinics and the hexoside isomers, the compounds were quantified based on the free form of the corresponding metabolite.

Phenolic compounds were extracted from both plasma and urine samples by solid phase extraction (SPE) as previously described by our research group with minor modifications [21]. Prior to the SPE, plasma and urine samples were acidified with *o*-phosphoric acid and formic acid, respectively, and urine samples were centrifuged at 11,884 rpm for 4 min at 4 °C. Then, MeOH (1 mL) and 1.5 M formic acid (1 mL) was added to activate the HLB plate 30 µm (30 mg). Plasma or urine sample (1 mL), previously acidified and spiked with ethyl gallate (IS), was loaded into the 96-well plate for clean-up with 1.5 M formic acid (1 mL) and 5% MeOH solution (1 mL). The elution was achieved with MeOH (1 mL) acidified with 0.1% formic acid. The elution fraction obtained was evaporated to dryness by a sample concentrator (Techne, Staffordshire, UK) at room temperature under a stream of nitrogen. 100 µL of water acidified with 0.1% formic acid was added to dissolve the residue and filtered through a 0.22 µm polytetrafluoroethylene (PTFE) syringe filters (Waters Corporation).

## 5. Conclusions

We have validated an UHPLC-MS/MS method to determine tomato phenolics and their metabolites in biological samples with a previous solid phase extraction capable of analyzing a high number of samples in a short period of time. To our knowledge, this is the first method reported for the rapid detection and quantification of tomato sauce phenolics and their microbiota-derived metabolites in plasma and urine samples. The method offers excellent sensitivity, reproducibility and recovery. This procedure, due to its rapidity and simplicity, can be applied in future clinical and epidemiological studies with a high number of blood and urine samples.

## Abbreviations

Acetonitrile (MeCN); collision-activated dissociation (CAD); declustering potential (DP); electrospray ionization (ESI); entrance potential (EP); ethanol (EtOH); focusing potential (FP); high performance liquid chromatography (HPLC); high performance liquid chromatography coupled to mass spectrometry in tandem mode (HPLC-MS/MS); internal standard (IS); limit of detection (LOD); limit of quantification (LOQ); mass spectrometry (MS); methanol (MeOH); multiple reaction monitoring (MRM); neutral loss (NL); polytetrafluoroethylene (PTFE); precursor ion scan (PrIS); product ion scan (PIS); relative standard deviation (RSD); retention time (RT); solid-phase extraction (SPE); ultra-high performance liquid chromatography (UHPLC); ultra-high performance liquid chromatography coupled to mass spectrometry in tandem mode (UHPLC-MS/MS).
